# Great Gray Owls hunting voles under snow hover to defeat an acoustic mirage

**DOI:** 10.1098/rspb.2022.1164

**Published:** 2022-11-30

**Authors:** Christopher J. Clark, James Duncan, Robert Dougherty

**Affiliations:** ^1^ Department of Evolution, Ecology, and Organismal Biology, University of California, Riverside, CA 92521, USA; ^2^ Discover Owls, Balmoral, Manitoba, Canada R03 0H0; ^3^ Department of Aeronautics & Astronautics, University of Washington, Seattle, WA 98195, USA

**Keywords:** acoustic camera, attenuation, playback, refraction, sensory ecology

## Abstract

How do Great Gray Owls (*Strix nebulosa*) capture voles (Cricetidae) through a layer of snow? As snow is a visual barrier, the owls locate voles by ear alone. To test how snow absorbs and refracts vole sound, we inserted a loudspeaker under the snowpack and analysed sound from the loudspeaker, first buried, then unburied. Snow attenuation coefficients rose with frequency (0.3 dB cm^−1^ at 500 Hz, 0.6 dB cm^−1^ at 3 kHz) such that low-frequency sound transmitted best. The Great Gray Owl has the largest facial disc of any owl, suggesting they are adapted to use this low-frequency sound. We used an acoustic camera to spatially localize sound source location, and show that snow also refracts prey sounds (refractive index: 1.16). To an owl not directly above the prey, this refraction creates an ‘acoustic mirage’: prey acoustic position is offset from its actual location. Their hunting strategy defeats this mirage because they hover directly over prey, which is the listening position with least refraction and least attenuation. Among all birds, the Great Gray Owl has the most extreme wing morphologies associated with quiet flight. These extreme wing traits may function to reduce the sounds of hovering, with implications for bioinspiration.

## 1. Introduction

In winter, predators like Great Gray Owls (*Strix nebulosa*) perform a remarkable feat: they catch voles (*Microtus* and *Myodes* spp.) hidden under a layer of obscuring snow [1]. They can take prey under snow up to 50 cm thick and punch through hardened crusts strong enough to hold a person's weight [2,3]. Here we examine: how do Great Gray Owls perform the difficult acoustic task of locating this prey through sound alone?

Laboratory experiments show some owl species can pinpoint both the azimuth and elevation of incoming sound to within 3° on account of their specialized ears [4–6]. For comparison, the human ear is less able to discriminate elevation [7]. Although owls exhibit amazing sound localization abilities under controlled laboratory settings, natural conditions may be more challenging. The environment may interfere with an owl's ability to locate prey, by distorting and attenuating sound on its path from vole to owl. Here we investigated an extreme form of environmental interference: snow.

Snow is a complex matrix of an ice skeleton and open-air pores. It has highly variable material properties, especially porosity and density. These material properties change with time, beginning as soon as a snowflake settles and chemically bonds with the snowpack [8]. Physical changes such as wind dramatically affect the snowpack, breaking apart individual snowflakes and redistributing and compressing snow in drifts across the landscape. Snow accumulates in layers [9]: a light, fluffy layer of fresh new snow may sit atop an older hardened layer. This dramatic variation in material and geometric properties presents a complex challenge to the owl and made it difficult for us to predict *a priori* precisely how the snow would affect sound.

Snow has multiple possible effects on sound. One effect is already familiar to anyone who has enjoyed the silence of a snowy landscape: snow absorbs sound. Attenuation coefficients for snow are on the order of 1 dB per cm [8] and vary with frequency and snow material properties [10].

Here we ask whether snow might refract sound enough to generate an ‘acoustic mirage’: a false impression of the location of the source of sound. Sound travelling through snow moves through both the air pores and the ice skeleton, at different speeds. Sound travelling through the ice skeleton often does so at higher speed, but may not be transmitted into the air, whereas the sound travelling through the air pores likely emanates out of the snow and is audible to the owl [8,10]. In air, the speed of sound (*c*) is defined by the relationship of pressure (*P*) and density (*ρ*) according to $c^{2}=\partial p/\partial \rho$ [11], where the partial derivative is taken according to the condition. In open air, this relationship is adiabatic: pressure and density exchange with little heat production. In this case, Laplace's hypothesis [11] gives $P=K\rho^{\gamma }$, where *K* is constant. The specific heat ratio $\gamma =c_{p}/c_{v}$ is 1.4 for air (where v is volume). The adiabatic speed of sound becomes $c=\sqrt{1.4\;P/\rho }$. When sound propagates in a dense porous medium such as open-cell (acoustic) foam, it primarily travels through viscous boundary layers. In this case, acoustic pressure change is affected by viscosity (per Poiseuille's law) and the process is instead isothermal. Then the ideal gas law with constant temperature establishes that P/ρ is constant and isothermal speed of sound becomes $c_{T}=\sqrt{\;P/\rho }$ [11,12]. Therefore we predicted that snow, as a dense porous medium, would have an index of refraction (*N*_snow_) of $\sqrt{1.4}\;=1.18$ [12]. Ishida [11] suggested that light-density snow might sit in between these two extremes (adiabatic or isothermal).

Here we ask: (i) How does snow distort and attenuate the acoustic cues available to a Great Gray Owl? We answer this question by conducting playback experiments with a loudspeaker under the snow, analysed with an acoustic camera, a device that produces images of the spatial distribution of sound sources, in addition to the spectral and temporal analysis provided by single-microphone systems. (ii) How do the available acoustic cues affect the strategy that Great Gray Owls use to hunt through snow? Owls generally ambush visible prey by flying straight towards the prey (e.g. supplementary videos in [13]), but Great Grays hunting through snow instead fly above prey and hover, then plunge straight down [1]. We created a geometric model of how snow distorts the sound as a function of owl position to test the significance of hovering over prey. (iii) Great Gray Owl is a morphological outlier [14]: why, out of all owls, does Great Gray Owl have the most extreme wing features associated with quiet flight? The answers to questions (i) and (ii) together imply that hovering above prey is the critical listening location that permits the owl to pinpoint prey location under snow. Thus, our results suggest that its extreme wing morphology may specifically function to reduce flight sounds during hovering flight.

## 2. Methods

### (a) Acoustics of snow

**Figure 1.**
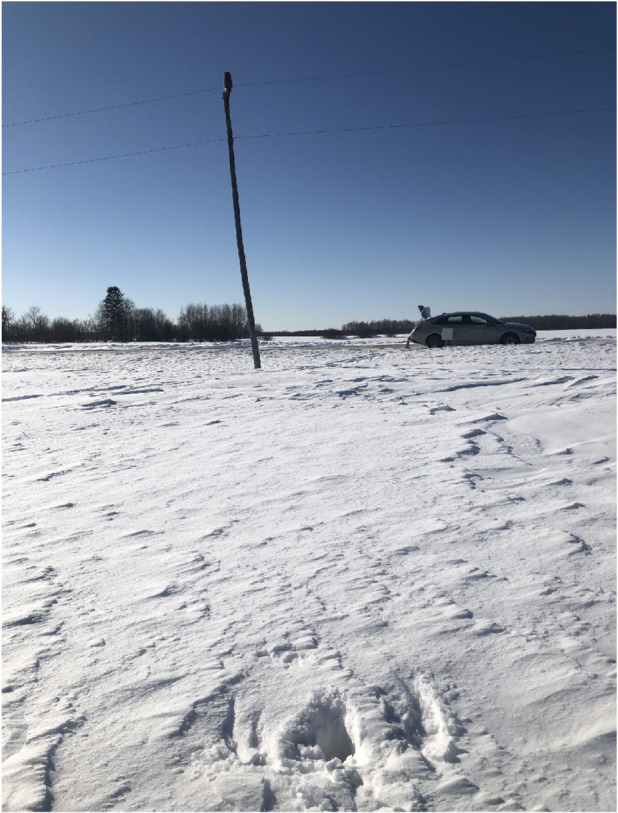
Fresh Great Gray Owl plunge hole (foreground), made by the owl perched on top of the telephone pole. Snow depth: 44 cm. Manitoba, Canada, 17 Feb 2022. See also electronic electronic supplementary material, figure S1. (Online version in colour.)

To test the acoustic effects of snow, in Manitoba, Canada (Feb. 2022) we located fresh plunge holes left in the snow after an attack (figure 1). On each hole, we measured snow depth and density with a snow-testing kit [15]. We dug a hole in the snowpack, then slid a waterproof subnivean loudspeaker (JBL Flip 5, Harman International) laterally into the subnivean layer at the bottom of the snowpack (average depth to top of loudspeaker: 0.40 m; table 1), so that there was intact snowpack between it and the acoustic camera. We noted the presence of a harder (icy) layer, created by a thaw in early winter, low in the snowpack in some of the holes, but we were not able to quantify its material properties. The acoustic camera was positioned 1.0–1.5 m vertically above the snowpack and horizontally between 1.2–6 m from the loudspeaker. We played white noise (equal amplitude between 0.1 and 20 kHz) interspersed with periods of silence. After recording the buried loudspeaker, we then dug away the snow to create a direct line of sight between the loudspeaker and camera, and again recorded the same white noise. In another experiment, we recorded a Meadow Vole (*Microtus pennsylvanicus*) digging under 12–15 cm of snow (see Electronic supplementary materials). These digging sounds were broadband and pulsed. In a single location we buried the loudspeaker and played back this sound, then successively removed layers of snow, such that we re-recorded these digging sounds with fixed acoustic camera and loudspeaker geometry at six different snow depths.

**Table 1.** Physical properties of the snow playback experiment. Means ± s.d. Snow depth measured from the surface of the snow to the ground; for owl-created plunge holes, prey depth was unknown (i.e. prey was not necessarily at the bottom of the snowpack).

snow	snow depth (m)	density (kg m^−3^)	sample size
loudspeaker playback	0.40 ± 0.06	234 ± 40	6
owl-created holes	0.50 ± 0.08	249 ± 32	7

### (b) Acoustic camera

The acoustic camera used was a SIG ACAM 100 Microphone Array (OptiNav Inc, Bellevue, WA, USA), run by a laptop computer with the software BeamformX, v. 6.008 (OptiNav, Inc). The acoustic camera consists of 40 MEMS microphones (InvenSense INMP441; flat frequency response [±3 dB]: 60 Hz – 15 kHz, minimum operating temperature: −40°C) arrayed in a spiral on a 0.4 × 0.4 m plate, and an optical camera in the centre. The microphones are sampled at 50 kHz (24 bit), unfiltered (Z-weighted). While the signal to noise ratio (SNR) of the individual microphones was 61 dB, this metric does not adequately capture the SNR of the bulk ensemble of microphones, on account of the beamforming. Instead the SNR is a function of recording conditions as measured *in situ* (see below).

The BeamformX software conducts beamforming, using the focal distance, ambient air temperature (−10 to −27°C) and frequency band (1/3 or 1/12 octave bandwidths) as inputs. For the selected bandwidth it calculates the microphone Cross Spectral Matrix (CSM). With a sufficient integration time (longer than 0.5 s) the off-diagonal elements of the CSM are dominated by acoustic sources, which are correlated among the microphones. Interfering noise sources such as microphone self-noise (the noise floor of individual microphones) cancel because they are uncorrelated between different microphones. The source locations and strengths are fitted to the CSM by an OptiNav-proprietary algorithm, Quantitative Beamforming [16,17]. The spatial locations of the sound source are painted as a ‘heat map’ onto a camera image (hereafter: the *acoustic position*) [18,19].

In the BeamformX software, we used the ‘region of interest’ (ROI) function to select a large area around the loudspeaker (see figures, which were cropped from the full optical camera image). The effect of the ROI is to include in the analysis only incoming sound from within this selected spatial region (i.e. from the area around the loudspeaker) and discard sound arriving at the camera from other sources directions. To be detected, sources within the ROI must be within 20–30 dB of the loudest sound around the camera. The noise floor was estimated by taking the spectrum from this ROI when the loudspeaker was not playing white noise since in that condition nearly all sound arriving at the camera will originate from outside the ROI.

We present values as Sound Pressure Level (SPL, ref. 20 µPa), which is subject to caveats. The camera was calibrated against a tonal sound source and returned values within ± 3 dB relative to a SPL metre. The following affect the accuracy of the numbers we present: the sounds analysed here are broadband (not tonal); the microphones are only flat down to 60 Hz; and although the microphones themselves are rated down to −40°C, whether the operating sensitivity of the camera as a whole is affected by temperatures below 0°C is not known.

We calculated attenuation coefficients for 1/12 octave bands, from the difference between sound levels of the buried and unburied loudspeaker, divided by the estimated distance for which sound travelled through the snow (in cm). We also estimated acoustic refraction index (*N*_snow_) from the shift in the centroid of the acoustic position of playback in buried versus unburied conditions, calculated by applying Snell's law of refraction to the geometry of the camera, snow and loudspeaker.

### (c) Acoustic model

We made a two-dimensional model of the acoustic problem that an owl faces, based on the depth (*D*) of the snow, the height (*H*) of the owl above the snow and the length (*L*) between the owl's current position and the true horizonal position of the vole. Assuming an *N*_snow_ of 1.2, we calculated two angles, which correspond to two potential owl attack strategies. If the owl seeks to determine the true elevation angle to the prey, refraction will cause the apparent (observed) angle of incoming sound to differ by an error angle, *γ*, from the true angle. Alternatively, the owl may seek the location that is on the surface of the snow, *vertically above* the vole, then intercept the vole by striking straight down from this point. For an owl that is not directly above the prey, the difference between the perceived elevation angle and the angle to the vertical position is the error angle *λ*. We compared these models to an exemplar video of a hunting Great Gray Owl published on the Internet (see electronic supplementary materials).

## 3. Results

The raw data associated with this study are in the electronic supplementary materials. Electronic supplementary material, figure S1 shows additional photographs of the experimental set-up.

### (a) Acoustics of snow: attenuation

**Figure 2.**
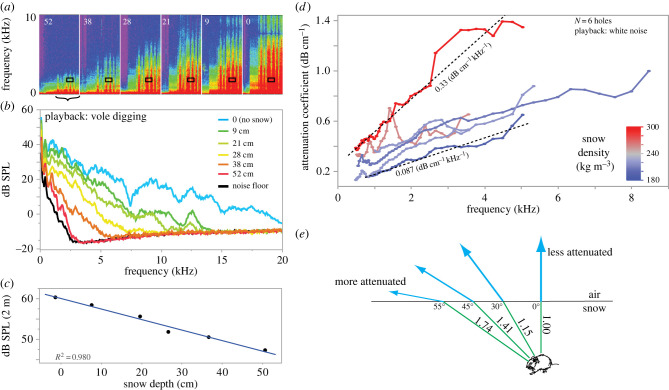
Snow attenuates sound, particularly at higher frequencies. (*a*) Spectrograms show playback of vole digging sounds. Depths of snow (cm) are given in white font on the upper left of each section. The black rectangles indicate the time span of sound analysed in (*b*), but not the frequency; (*b*) shows the entire bandwidth of sound. The rectangles indicate both the time frame and spectrum (97 Hz band) shown in figure 3*a*. Spectrograms show sound from within the region of interest (ROI) of the acoustic camera, corresponding to images shown in figure 3*a*. (*b*) Power spectrum of 1 s of sound (48.8 Hz bins) from within the ROI (figure 3*a*). Black: no playback, i.e. showing the acoustic camera noise floor (background). (*c*) Overall Sound Pressure Levels (SPL) (calculated as the sum of amplitudes from power spectra in (*b*) recorded at the acoustic camera, 2 m from the loudspeaker, as a function of snow depth. Slope: −0.26 dB per cm, regression, *p* = 0.0002. (*d*) Attenuation coefficient from analysis of white noise (lowest and highest slopes indicated, assuming a linear fit). Calculated from the excess attenuation (the difference in peak levels between snow-covered and snow-uncovered loudspeakers), divided by distance travelled through snow in cm. Results derived from acoustic camera output: acoustic peak (1/12 octave bands, 0.5–10 kHz). There is a positive correlation between density and attenuation coefficient (2 kHz only, linear model, *p* = 0.017; covariate: distance through snow, *p* = 0.11, *N* = 6). (*e*) Snow attenuates sound in proportion to the distance it travels through snow. Therefore, sound emanating normal (0°) from the prey is least attenuated (blue vectors), while higher angles travel a greater distance through snow and have greater snow-induced attenuation. E.g. Sound emanated at *θ* = 55° travels 1.74 times the distance of sound at 0°. Angles drawn assuming *N*_snow_ = 1.2 (figure 3). Data shown here are supplied in the electronic supplementary material. (Online version in colour.)

A vole digging under 15 cm of snow produced broadband, pulsed sounds such that, at 0.6 m above the snow, the overall SPL of 1 second of sound was 25.2 dB (bandwidth: 0.2–10 kHz). Playing back this vole digging sound (at 60 dB SPL, ref. 2 m) under snow indicated that snow dramatically attenuated sound (figure 2*a–c*). This attenuation was a function of snow depth (figure 2*c*) and the greatest attenuation occurred at high frequency (figure 2*a,b*). Across the full spectrum, the vole digging sounds attenuated at −0.26 dB cm^−1^ (figure 2*c*). White noise was played back with an SPL level of 68.5 dB (ref. 2 m; electronic supplementary material, figure S2) and snow attenuation coefficients varied with frequency and snow density (Electronic supplementary material, figure 2D). Across six holes, which had similar depths and densities to the snow of actual Great Gray Owl plunge holes (table 1; electronic supplementary material, figure S1), the attenuation coefficient varied between 0.1–0.4 dB cm^−1^ at 0.5 kHz, rising to 0.6–1.3 dB cm^−1^ at 5.0 kHz. Moreover, higher-density snow tended to have greater attenuation coefficients (electronic supplementary material, figure 2D). In none of the holes could we calculate attenuation coefficients at frequencies of 10 kHz or above, because so much sound was absorbed at higher frequencies that there was insufficient signal from the buried loudspeaker, given the sensitivity of the acoustic camera.

### (b) Acoustics of snow: refraction

**Figure 3.**
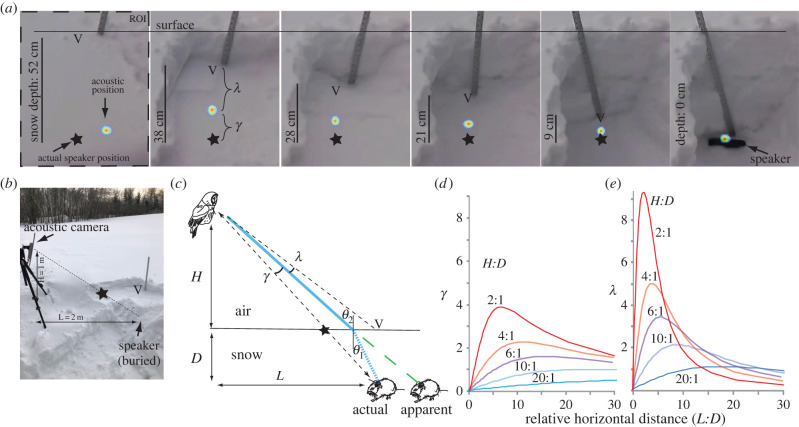
Snow refracts sound, thus at certain angles the owl perceives an ‘acoustic mirage’: the acoustic position is offset from the actual source position. (*a*) The acoustic position of sound (heat map) changes with snow depth, due to refraction. V: vertical location above loudspeaker. Star: actual location of loudspeaker. Beamforming settings: 1/3 octave bandwidth centred on 2.0 kHz, 1 second integration time (beamforming corresponds to black rectangles in figure 2*a*). Images have been cropped from the full camera image to show the ROI as defined in the BeamformX software. (*b*) Experimental set-up; snow depth = 38 cm (top 14 cm of snow already removed). (*c*) Geometry of the model: *L*: length of the horizontal distance from sound source. *D*: depth of the sound source below snow. *H*: height above snow. V and star indicate two possible attack strategies. Blue line shows path of sound refracted at the air–snow interface; green dashed line shows apparent location. (*d,e*) Error angles *γ* and *λ* as a function of *L*, *D*, and *H*. Data shown in (*a*) correspond to *H*:*D* = 2.6, *L*:*D* = 5.2. The actual hunting strategy of Great Gray Owls is to approach prey at *H*:*D* ratios of > 10:1 (e.g. figure 4). (Online version in colour.)

Playback of white noise, analysed with the acoustic camera, showed that in nearly all trials, the acoustic position of inbound sound was shifted in a direction consistent with refraction (figure 3*a,b*; *N* = 6 holes). The presence of snow shifted the acoustic position between 1.7 and 4.7 degrees (*γ*) relative to the acoustic position of the unburied loudspeaker. Accounting for geometry differences among trials, and assuming the snow surface was flat with constant snow material properties, yielded an estimate of *N*_Snow_ = 1.16 (mean; range: 1.05–1.27), with no correlation between *N*_snow_ and snow density (regression, *N* = 6 samples, *p* = 0.51), close to our predicted value of 1.18. In certain circumstances (e.g. in pilot data not shown), the acoustic position corresponded to other acoustic effects, including reflections (e.g. if we didn't backfill the hole), and other idiosyncratic shifts in acoustic position associated with poor SNR. For instance, the acoustic position at a depth of 52 cm in figure 3*a* is not shifted consistent with refraction, while the acoustic position in the other panels of figure 3*a* (depths 38, 28, 21, and 9 cm) are consistent with refraction.

In our model of refraction (figure 3*c*), distances L and H are non-dimensionalized by expressing them as ratios of snow depth, *D*. Both error angles *γ* and *λ* converge on 0 when L:D = 0; and are > 0 in any other position (figure 3*d,e*). For both angles, the height of the owl above the snow had substantial effect on the magnitude of the angle. For an owl two snow-depths above the snow (H:D = 2:1), when L:D was roughly 3 to 5, *γ* and *λ* approach maximum values of 4° and 10°. By contrast, for an owl 20 snow-depths above the snow (H:D = 20:1), neither angle ever exceeds 1° (figure 3*d,e*). Thus, irrespective of which exact strategy the owl employs (i.e. whether the owl tries to minimize *γ* or *λ*), the approach is the same: the owl will best be able to pinpoint the location of the prey if it listens from overhead from several snow-depths above the surface of the snow.

### (c) Owl hunting behaviour

**Figure 4.**
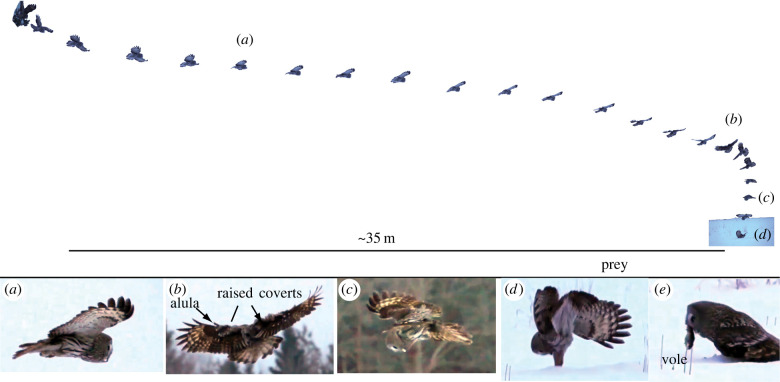
Kinematics of a wild Great Gray Owl (*Strix nebulosa*) successfully hunting a vole in Finland. Images are from electronic supplementary material, video S1. (*a*) Glides: the owl glides from its perch (at roughly 6.3 m s^−1^) until it is over the vole. (*b*) Hovers: when the owl arrives in the vicinity of prey it slows and turns; the alula is raised and elevated, and fluttering upper wing covert feathers (see electronic supplementary material, video S1) imply stall over portions of the wings. (*c*) Parachutes: the owl descends nearly straight down at approximately 4.5 m s^−1^, with head pointed down. (*d*) Strike: just before reaching the ground, the owl thrusts its legs down. Snow depth appeared to be 20–30 cm. (e) Eventually the owl transfered a vole (*Microtus agrestis*) to its beak. Video courtesy of Sylvan Eckhardt. (Online version in colour.)

An exemplar of a successful hunt, electronic supplementary material, Video S1, shows four stages: gliding (figure 4*a*), hovering (figure 4*b*), parachuting (figure 4*c*) and the strike (figure 4*d*). In this exemplar the owl only hovered momentarily, while literature reports of attacks suggest hovering can last up to 10 s [20]. During hovering, videos revealed extensive aerodynamic stall on the wings (figure 4*b*, electronic supplementary materials), as evidenced by raised, fluttering upper wing coverts; the alula (thumb-feather) was also raised.

## 4. Discussion

Our playback data begin to unpack the nature of the formidable acoustic challenge confronted by a predator when it tries to use sound to locate prey hidden under a layer of obscuring snow. At least three environmental effects of sound transmission influence the acoustic cues available to the owl.

First, sound attenuates with distance. Great Gray Owls launch attacks from as far as 50 m (horizontal distance) away (figure 3, [21]). Assuming they launch an attack upon hearing sound, how faint are the sounds they can hear at this great distance? While we have no data on the actual sound levels produced by prey in the wild, a vole digging under 15 cm of snow under artificial conditions had sound levels attenuated to roughly 20 dB SPL at 1 m distance. Assuming spherical spreading, this sound would attenuate to −9.5 dB at 30 m or −14 dB at 50 m distance. Several other owl species have auditory thresholds near −9 dB [22], although thresholds of the Great Gray have not been measured. An owl hearing a faint sound just above its detection limits has limited ability to localize sound [23]. To localize a distant source, it will need to listen as it approaches and perform in-flight course corrections, as Barn owls have been shown to do experimentally [24]. Observations of Great Gray Owl re-orienting during midflight suggest they may do this as well [2].

**Figure 5.**
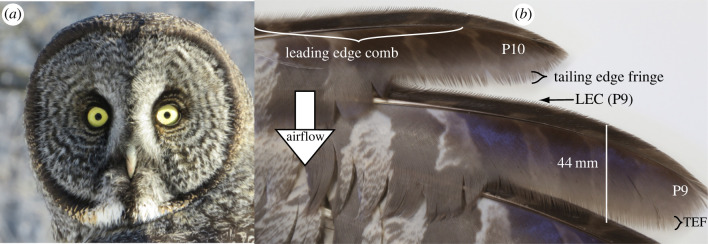
Extreme morphological features that the Great Gray Owl uses to hunt through snow. (*a*) This species has the largest facial disc of any owl [14], where this ring of feathers filters and amplifies sound at the ears. The large size of the facial disc suggests that Great Gray Owls listen to low-frequency sound. (*b*) Ventral view of two traits associated with quiet flight in outer wing feathers, P10 and P9. The leading edge comb (LEC) extends out from the front edge of the wing and, in P10, is up to 6 mm long, while P9 has a shorter LEC (black arrow). The trailing edge fringe (TEF) is a wispy margin extending from the edge of the vane and in these feathers is also up to 6 mm long. Photo is of specimen (UWBM #39063) from the Burke Museum, Seattle, WA. (Online version in colour.)

Second, snow dramatically attenuates sound (figure 2). The effect is much stronger at high frequencies, with little sound above 3 kHz escaping from deep snow, even at high playback source amplitudes (figure 2*b*). Barn owls (*Tyto alba*) use inter-aural level differences of sounds between 3 and 8 kHz to localize prey elevation [25], suggesting that owls hunting prey under snow may have reduced ability to determine prey elevation. The Great Gray Owl has the largest facial disc of any owl [14] (figure 5*a*). As size sets the lower frequency of sound that this disc filters, the large size of their disc implies that the Great Gray is specialized for hearing low-frequency sound.

Moreover, the attenuation of sound by a porous medium such as snow is directional [26]. The shortest path through the snow is straight up; sound that propagates at an angle passes through more snow before emerging into the air, and thus experiences more attenuation by snow (figure 2*d*). This effect implies that the best place to listen for prey is from directly overhead, where the sound transmitting through the snow is least attenuated, and it also explains why Great Gray Owls tend to hunt by listening for prey from a perch high (often 5 m or more) above the snow.

Third, snow also substantially refracts sound (figure 3). From any position other than overhead, refraction will interfere with the owl's ability to pinpoint the prey's exact location. This effect is exacerbated if the bird is low, just above the surface of the snow (figure 3*d,e*). Hovering overhead (figure 4*b*) puts the owl in the location that alleviates the effect of refraction. This is the same strategy that many plunge-diving birds take when hunting through the air–water interface, where light passing from water to air is refracted. Osprey, kingfishers, gannets [27] and some herons [28] strike at underwater prey straight down or nearly so, an orientation that minimizes refraction. By contrast, certain heron species can strike through the air–water interface at an angle because they can account for refraction [28], as do archerfish that spit water at insects when their eyes are completely underwater [29]. Archerfish or herons can see their prey, thus these predators are able to estimate the true strike distance and can compensate for refraction (i.e. take into account angle *γ*). By contrast, a predator hunting prey hidden under snow will always have uncertainty about how deep its prey is. The owl could possibly estimate depth by attending to how ‘muffled’ the sound seems: i.e. the excess attenuation of higher frequencies that snow causes (figure 2). However, this will be an imperfect cue of depth, because attenuation also varies substantially with snow density (figure 2*c*), which is also unknown to the owl. Therefore, it is unclear whether the owl may have enough information to accurately calculate *γ*.

We have focused on the predator in this predator–prey interaction. What about sounds transmitted to a vole under the snow? Refraction of sound travelling from air into the snow will tend to produce an acoustic version of Snell's window: since the critical angle (given *N*_snow_ = 1.2) is 56.4°, all sound arriving at the animal's location under the snow will arrive within a cone of about 113° centered overhead. Given that snow attenuates these sounds as well, and the hearing of subnivian prey such as voles is not well studied, whether animals under the snow attend to sounds is unclear.

### (a) Acoustics of hovering

These environmental sound-transmission effects all suggest that the critical spot from which the owl can pinpoint the position of prey is to listen from directly overhead when the owl is hovering. This result has two implications for another way in which Great Gray Owl is extreme: quiet flight.

Among all owls, the Great Gray seems to be a quiet flight extremist (figure 5). It has among the longest leading edge comb [14], the thickest velvet [30,31], and it might have the longest and most extensive vane fringes of any owl (figure 5), although this last wing feature has not yet been the subject of a careful interspecific comparison. These wing traits all apparently reduce flight sounds, although the form–function relationship of these traits, i.e. how changes in length of the comb or vane fringe affect sound levels in flight, remains incompletely understood [31,32]. Assuming these traits are related to the Great Gray Owl's specialization for hunting through snow, what is the significance of the large size of these traits in the Great Gray Owl?

There are at least two possible reasons. First, since snow absorbs high-frequency sound, the most salient sounds available for a Great Gray Owl to use are below 3 kHz (figure 2). Therefore, perhaps the size of the quieting features on Great Gray Owl wings suppress a lower-frequency spectrum of sound than the quieting features of other owls, thereby permitting it to hear better in this frequency range. Second, the large size of these wing traits may specifically ameliorate sounds produced during hovering, rather than other modes of flight, since our hypothesis is that the critical position to listen and pinpoint prey location is when hovering overhead. Although certain aspects of quiet flight have been studied extensively, the role of flight kinematics in this predator–prey interaction is both clearly critical to the sound that the owls' wings produce, and remains virtually unexplored [31]. During hovering, the alula (thumb-feathers) is raised and covert feathers over parts of the wing are raised and fluttering, implying that, in hovering, these parts of the wing are at or close to aerodynamic stall (e.g. figure 4, electronic supplementary material, video). As it happens, other birds that hunt by hovering above prey obscured under grass while attempting to pinpoint their location include American Kestrel (*Falco sparverius*), harriers (*Circus* spp.) and kites (*Elanus* spp.). All of these species have convergently evolved the same velvet coating on their wings as owls [30,33], suggesting that the acoustics of hovering has selected for features that dampen flight sounds. Investigating whether and how the extreme wing features of Great Gray Owl wings affect sound production *in hovering flight* may be a fruitful and unexplored future avenue for bioinspiration of quiet flight.

## Data Availability

The data presented in the paper have been uploaded as a zipped folder in the electronic supplementary material [34].
